# Development of Highly Sensitive Strain Sensor Using Area-Arrayed Graphene Nanoribbons

**DOI:** 10.3390/nano11071701

**Published:** 2021-06-28

**Authors:** Ken Suzuki, Ryohei Nakagawa, Qinqiang Zhang, Hideo Miura

**Affiliations:** Fracture and Reliability Research Institute, Graduate School of Engineering, Tohoku University, 6-6-11 Aoba, Aramaki, Aoba-ku, Sendai 980-8579, Japan; miuralab-web@rift.mech.tohoku.ac.jp (R.N.); zhang.qinqiang@rift.mech.tohoku.ac.jp (Q.Z.); hmiura@rift.mech.tohoku.ac.jp (H.M.)

**Keywords:** graphene nanoribbon, piezoresistivity, strain sensor

## Abstract

In this study, a basic design of area-arrayed graphene nanoribbon (GNR) strain sensors was proposed to realize the next generation of strain sensors. To fabricate the area-arrayed GNRs, a top-down approach was employed, in which GNRs were cut out from a large graphene sheet using an electron beam lithography technique. GNRs with widths of 400 nm, 300 nm, 200 nm, and 50 nm were fabricated, and their current-voltage characteristics were evaluated. The current values of GNRs with widths of 200 nm and above increased linearly with increasing applied voltage, indicating that these GNRs were metallic conductors and a good ohmic junction was formed between graphene and the electrode. There were two types of GNRs with a width of 50 nm, one with a linear current–voltage relationship and the other with a nonlinear one. We evaluated the strain sensitivity of the 50 nm GNR exhibiting metallic conduction by applying a four-point bending test, and found that the gauge factor of this GNR was about 50. Thus, GNRs with a width of about 50 nm can be used to realize a highly sensitive strain sensor.

## 1. Introduction

In an aging society with a declining birthrate, the development of structural health-monitoring systems is extremely important in order to maintain a secure and reliable society for human beings through early detection of material damage. Especially in basic infrastructures such as energy plants and transportation systems, online damage-monitoring technology is strongly required due to the complexity of the systems and the shortage of skilled engineers due to the declining birthrate and aging population. In order to quantitatively evaluate material damage and prevent various failures, it is necessary to monitor the stress and strain fields of components in operation with high sensitivity and low cost; in other words, there is a strong demand for highly sensitive strain sensors. For these needs, strain sensors based on carbon nanoparticles, such as carbon nanotubes and graphene, have attracted attention as a viable alternative to conventional sensors based on metallic or semiconducting materials, mainly due to their superior electrical properties. 

Various sensors based on carbon nanomaterials have been proposed and practically used, and most of them are made by adding carbon fillers to a polymer matrix and forming an electrical network between the carbon fillers to provide high piezoresistivity [1,2,3,4,5]. Although these sensors have excellent flexibility, processability, and repeatability, there still exist difficulties in achieving high strain sensitivity and high resolution by reducing the size of the sensor element. In particular, as materials become more multi-elemental, composite, and miniaturized to improve their performance, strain (stress) distribution within the material increases due to compositional fluctuations and mismatch of structure and physical properties between different materials, leading to not only local damage, but also final fracture. Therefore, in damage monitoring, it is important to monitor not only the maximum value of strain (stress), but also its spatial distribution. 

Recently, much attention has been focused on graphene as an available material for strain sensors with high sensitivity and large deformability [6]. Graphene is a monolayer of graphite possessing a honeycomb-like unit cell of carbon atoms. It exhibits high intrinsic strength around 130 GPa, large deformability over 20%, and high electron mobility above 2.0 × 10^5^ cm^2^ V^−1^ s^−1^ [7,8,9,10]. In general, graphene sheets are good conductors; however, when graphene is cut into thin ribbons with nanoscale widths, called graphene nanoribbons (GNRs), the GNRs begin to exhibit semiconducting properties. GNRs are classified into two groups according to the structure of their ribbon edge: armchair GNRs (AGNRs) and zigzag GNRs (ZGNRs) [11]. ZGNRs form a local electron orbital distribution around the zigzag edge due to the quantum confinement effect, and exhibit metallic electronic properties regardless of the ribbon width [12]. On the other hand, AGNRs have been theoretically shown to exhibit semiconducting or metallic properties depending on the width, defined by the total number of dimer chains of carbon atoms in the width direction, as well as significant strain sensitivity in electronic band structure and electronic transport properties [13,14,15,16]. Therefore, the development of strain sensors that can detect micro-strains by using GNRs with their large piezoresistive effect has great potential.

In this paper, we propose a basic design of an area-array-type, GNR-based strain sensor that can measure micro-strains with high sensitivity and high resolution. Each GNR arranged in an area array works as a strain gauge, and thus the distribution of strain in the area to be measured can be evaluated. In order to realize a GNR-based high-sensitivity strain sensor, it is essential to fabricate a high-quality GNR stably. High-quality GNRs can be fabricated by two methods: the bottom-up method and the top-down method. The bottom-up method is a direct synthesis of GNRs using synthetic chemical methods. The remarkable advantage of the bottom-up approach is the accuracy in controlling the ribbon width, edge structure, and film thickness when forming thin films at the atomic level, enabling accurate GNR structures with angstrom-level resolution and obtaining physically smooth and chemically clean edges [17,18,19]. However, it is difficult to combine this method with conventional thin-film device-fabrication processes, such as photolithography and deposition processes, and to attach electrodes to the synthesized GNRs for device applications. On the other hand, the top-down method is to cut out GNRs by etching a large graphene sheet using reactive ion etching (RIE) or other methods [20,21,22]. Since this method can be combined with the conventional thin-film device-fabrication process, and provides more flexibility in the design of GNR width and length than the bottom-up method, GNRs were fabricated by the top-down method in this study. The proposed strain sensor can not only take advantage of GNR’s properties, such as high strain sensitivity and high strength, but also can be applied to various applications such as the sensing part of industrial robots and sensors for human health monitoring, because it is easy to design spatial resolution and miniaturization by the top-down method. To demonstrate the possibility of developing the proposed area-array-type, GNR-based strain sensor, we fabricated a two-dimensional array of GNRs on a Si substrate with 1 mm spacing and evaluated the quality and basic electronic properties of the fabricated GNRs.

## 2. Fabrication of Area-Aligned Graphene Nanoribbons

In order to establish a stable and reliable fabrication process of the device using GNRs, the synthesis of a high-quality, single-layer graphene sheet is one of the important factors. In this study, graphene was grown on the inner surface of a Cu foil pocket structure by applying thermal LPCVD (low-pressure chemical vapor deposition) using acetylene [23]. Since the dissociation energy of acetylene is lower than that of methane, which is often used as the source gas for the graphene growth, the growth rate of acetylene-based LPCVD is much faster than that of the conventional methane-based LPCVD, resulting in a lower production cost. However, the low dissociation energy of acetylene induces the formation of amorphous or multilayer graphene. Thus, in order to synthesize a high-quality graphene sheet by suppressing the formation of amorphous and multilayer sheets, it is necessary to control the supply of acetylene molecules on the surface of copper to avoid excessive supply [24,25]. Thus, the Cu pocket technique, which involves folding the Cu foil into a pocket structure with the remaining three sides carefully crimped with a pincer [26], was employed. Then, graphene was synthesized on the prepared Cu foil using acetylene and hydrogen at 1035 °C. The flow rates of acetylene and hydrogen were 80 sccm and 350 sccm, respectively, and the growth time was 30 min. 

**Figure 1:**
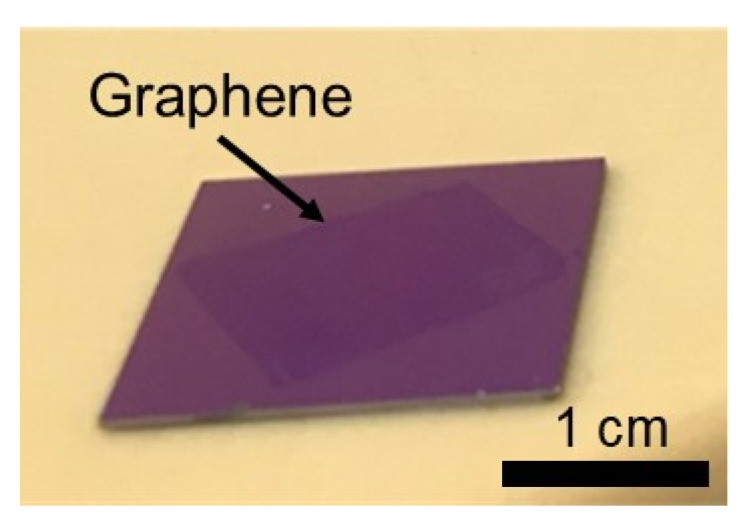
Optical image of graphene on a silicon substrate.

In order to fabricate a GNR structure, the graphene synthesized on the Cu foil was transferred onto an oxidized silicon substrate. In this study, a wet transfer process was applied using PMMA (polymethyl methacrylate). This transfer process is also known as the PMMA-assisted transfer process [27]. At first, the surface of the graphene sheet was covered with PMMA, and then the copper substrate was chemically etched off with FeCl_3_. After washing the etched sample with dilute HCl and pure water, the cleaned sample was placed on a silicon substrate with a 300 nm thick thermal oxide film, and the PMMA layer was removed with acetone. An example of the transferred graphene is shown in Figure 1. 

**Figure 2:**
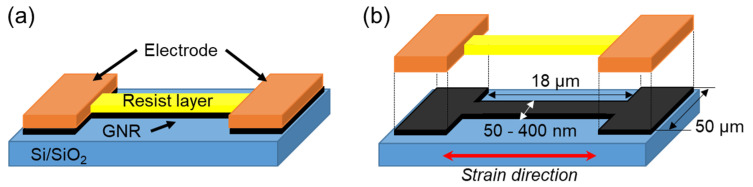
(**a**) Schematic image of the strain gauge in the area-arrayed GNR strain sensor, and (**b**) GNR structure of the strain gauge.

Figure 2 shows the schematic image of the GNR-based strain sensor fabricated in this study. The electrodes, Pt and Au, were deposited on a large area of graphene, and the GNRs for the sensor gauge were fabricated between the electrodes by electron beam (EB) lithography. The resist layer, which was used as a mask during etching, was deposited on the GNRs as an environment-resistant protective film. The contact resistance and Schottky barrier at the interface between different materials must be considered when electrically connecting different materials to form a device. In particular, when GNRs are used as strain sensors, it is necessary to realize good ohmic contact between GNRs and metal electrodes. As shown in Figure 2b, the electrode thin films were bonded with graphene of the same size as the metal electrode (electrode graphene), and the electrode graphene and GNR were seamlessly connected, so that the Schottky barrier could be reduced. 

**Figure 3:**
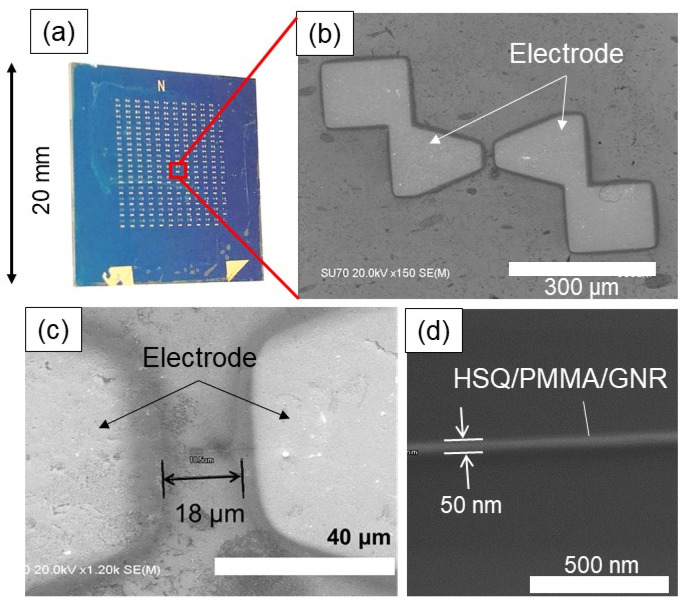
Observation of the -arrayed GNRs: (**a**) electrode arrangement, (**b**) SEM image of electrodes, (**c**) SEM image of gauge (GNRs), (**d**) magnified image of GNR with a width of 50 nm.

The patterning process using EB lithography is described as follows. After the graphene was transferred onto the Si substrate, a Pt layer with a 5 nm thickness and a Au layer with an 80 nm thickness were deposited continuously by EB deposition using a stencil mask to form metal electrodes. After deposition of the metal electrodes, GNRs were patterned between the electrodes using the EB lithography technique. First, PMMA was coated and cured on graphene, and then HSQ (hydrogen silsesquioxane: XR1541-006 Toray Dow Corning) was spin-coated as a negative resist to form an HSQ/PMMA laminated resist layer. As PMMA has a high affinity for graphene, sufficient adhesion can be achieved with low-temperature curing compared to the case of a single layer of HSQ. Next, line patterns ranging from 50 nm to 400 nm in width and 18 μm in length were irradiated onto HSQ and developed with TMAH (tetramethylammonium hydroxide) to form the patterns. During the development, the PMMA layer was not dissolved in TMAH, and only HSQ was developed. Finally, PMMA was removed by RIE with O_2_ plasma. The etching rate of PMMA with O_2_ plasma was 30 to 40 times larger than that of HSQ, and thus the HSQ pattern could be transferred to the graphene layer [28]. An example of the area-arrayed GNRs is shown in Figure 3. The composite structure of graphene with GNRs ranging from 50 to 400 nm in width was successfully formed under the HSQ/PMMA layer and electrodes.

## 3. Results and Discussion

### 3.1. Evaluation of the Quality of Fabricated Graphene Nanoribbons

**Figure 4:**
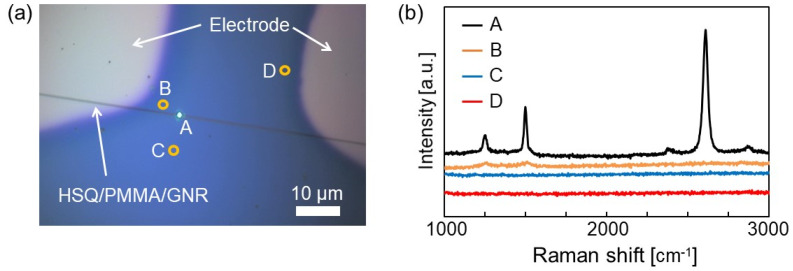
(**a**) Measurement points for Raman spectrum data. The white area in the figure is the metal electrode, and the black line in the center of the figure is the HSQ/PMMA/GNR. (**b**) Raman spectra of the indicated measurement points; A is the HSQ/PMMA/GNR layer, and B, C, and D are etched areas.

The quality of the fabricated GNRs was evaluated by Raman spectroscopy. When a laser beam is irradiated on the surface of graphene, three typical Raman shifts are known to appear in the resulting reflection spectrum. One is called the G band, which appears at 1590 cm^−1^ and corresponds to carbon–carbon bonds; the second peak is called the D band, which appears at 1350 cm^−1^ and indicates the presence of disordered carbon atoms, or defects. The last peak, called the 2D band, appears at 2700 cm^−1^ and is related to the total number of layers in the graphene sheet. The relative intensity of the D and G bands (I_D_/I_G_) corresponds to the defect density in the observed region, and the relative ratio of I_2D_ to I_G_ corresponds to the number of layers in the graphene sheet. Therefore, the quality of graphene sheets improves with decreasing I_D_/I_G_ values, and if the I_2D_/I_G_ value is greater than 2.0, the graphene is considered to be composed of a single layer. For the fabricated GNRs, Raman spectroscopy was used to verify whether graphene existed under the resist layer and whether the etching removed the unused graphene. As shown in Figure 4a, Raman spectra were obtained from different locations around the sample (400 nm width) etched under oxygen plasma using the RIE method. Figure 4b shows the obtained Raman spectra. At the measurement point A in the patterned region, three peaks originating from the graphene structure were observed, confirming the presence of the graphene layer. The I_2D_/I_G_ ratio was approximately 2.6, which indicated the presence of single layer of graphene. On the other hand, the intensity of the D band increased compared to graphene before the GNR fabrication process, with the ID/IG ratio increasing from 0.09 [21] to 0.4. At measurement points B, C, and D in the figure, the absence of graphene on the substrate was clearly confirmed. These data indicate that the patterning of the GNR structure was successfully achieved.

### 3.2. Electronic Characteristics of Fabricated Graphene Nanoribbons

**Figure 5:**
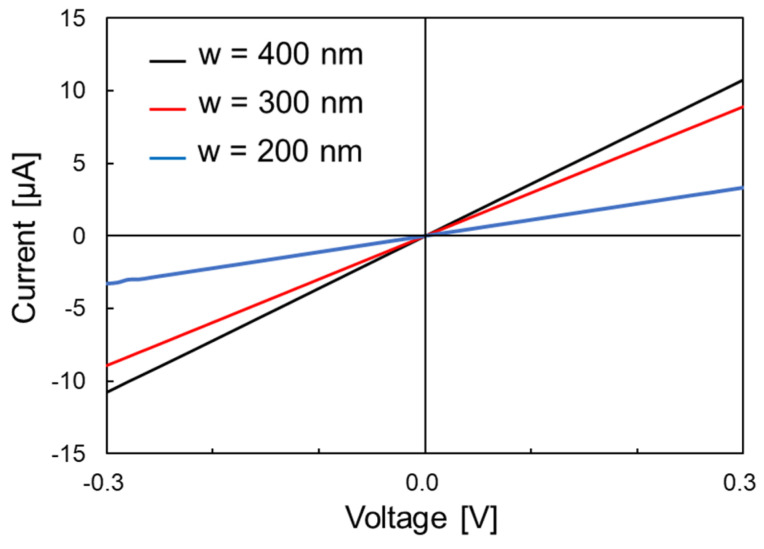
I–V characteristics of GNRs with widths (w) of 400, 300, and 200 nm.

Figure 5 shows an example of the I–V characteristics of GNRs with widths of 400 nm, 300 nm, and 200 nm. The measurement was performed using a two-point probe method at room temperature under an atmosphere environment. For all GNRs, the current value was found to increase linearly with increasing voltage. This confirmed that the fabricated GNRs with widths of 400 nm, 300 nm, and 200 nm exhibited metallic electrical properties, and a good ohmic contact was realized between graphene and the electrode, the Au/Pt thin film. The sheet resistance of the GNRs formed on the sample chip was evaluated from the I-V measurement results. Three GNRs each were measured for GNRs of 400 nm, 300 nm, and 200 nm widths formed on the same substrate. It is known that there is a correlation between graphene quality and sheet resistance, with higher-quality graphene having lower sheet resistance. For example, the sheet resistance of high-quality graphene with I_D_/I_G_ < 0.05 sold by BGT Material Limited is 300–600 Ω/square [29]. The sheet resistances of the 400 nm-wide GNRs were 600 Ω/square at maximum and 560 Ω/square at minimum, and those of the 300 nm-wide GNRs were 750 Ω/square at maximum and 510 Ω/ square at minimum, which were close to that of high-quality graphene sheets. The sheet-resistance values of almost all the fabricated GNRs did not change significantly before and after the GNR fabrication process, indicating that the amount of damage caused by the fabrication process was insignificant. On the other hand, for the GNRs with a width of 200 nm, the average sheet resistance increased to 1000 Ω/square. This was thought to be due to the increase in the fraction of area affected by side etching as the width decreased. Near the edge of the GNR width direction, the crystal quality was degraded by side etching and the electrical conductivity was reduced. The length of this side etching area and the resulting degradation in quality was expected to be almost constant regardless of the pattern width. Therefore, as the width decreased, the fraction of low-quality regions increased, resulting in an increase in sheet resistance.

**Figure 6:**
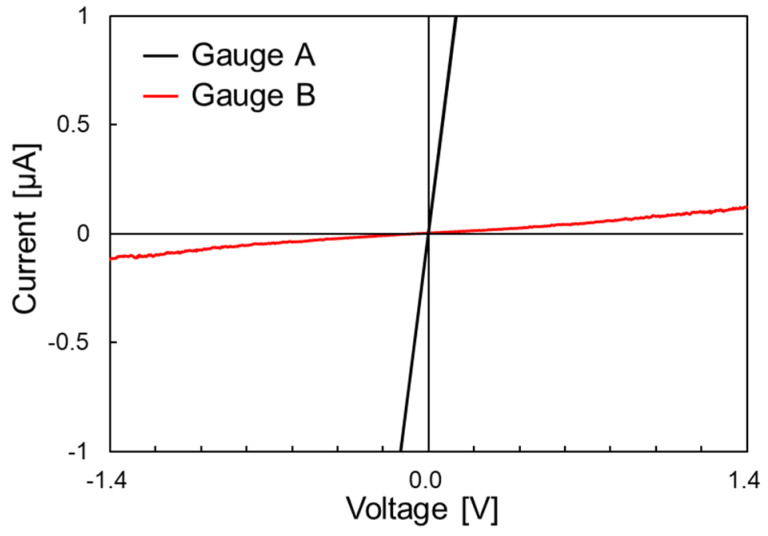
I–V characteristics of GNRs with a width of 50 nm.

Figure 6 shows an example of the I–V characteristics obtained for a GNR with a width of 50 nm. For GNRs with a width of 50 nm, there were two types of GNRs: a GNR in which the current increased linearly with increasing applied voltage (gauge A) and a GNR in which the current increased nonlinearly (gauge B). As shown in Figure 5, good ohmic contact was achieved between the metal electrode and graphene, and the difference in electrical conductivity likely was due to the different properties of the two fabricated GNRs, rather than the metal–graphene contact. The sheet resistance of gauge A was about 300 Ω/square, which was equivalent to that of a high-quality graphene sheet. On the other hand, the resistance of gauge B was very large, and the linear relationship between voltage and current was found to be slightly broken. Theoretical analysis has shown that a band gap appears in AGNRs with nanometer widths of less than about 100 nm [20]. Although the edge structure was not confirmed in this study, there was a possibility that the band gap was opened by decreasing the ribbon width to 50 nm, resulting in the GNRs exhibiting semiconducting properties. This implied that GNRs with a ribbon width of 50 nm exhibit a piezoresistive effect due to the quantum size effect.

### 3.3. Evaluation of the Strain Sensitivity of the Fabricated Graphene Nanoribbons

**Figure 7:**
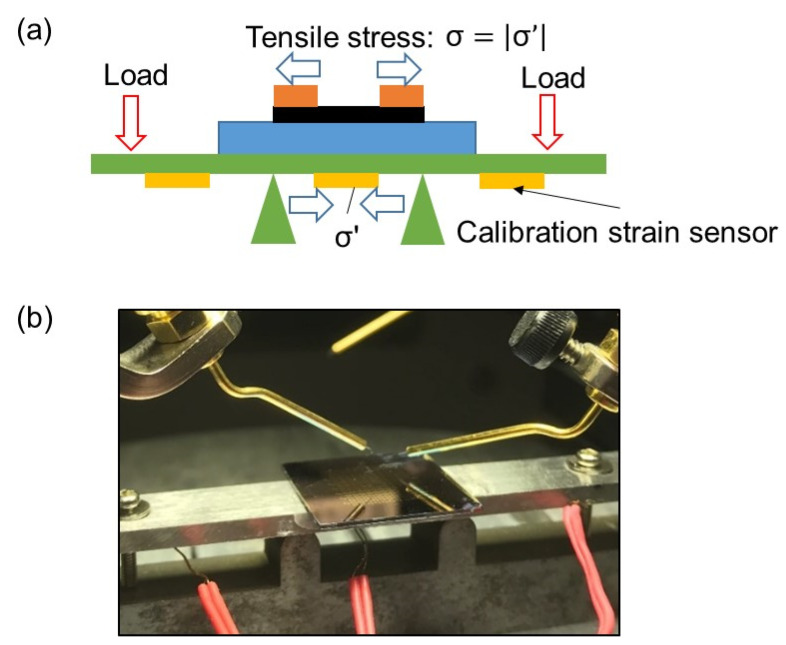
(**a**) Schematic of the four-point bending test principle, and (**b**) test setup.

Since it has been theoretically shown that AGNRs exhibit piezoresistive effects regardless of whether their initial electrical conduction properties are metallic or semiconducting [30], it was expected that the 50 nm-wide GNRs fabricated in this study would exhibit piezoresistive effects. Therefore, in order to confirm that the 50 nm-wide GNRs had high strain sensitivity, the strain dependence of the electrical conduction properties of the GNR with a width of 50 nm, which showed metallic conduction properties as shown in Figure 6 (gauge A), was evaluated using a four-point bending test. A schematic of the four-point bending test principle and test setup is shown in Figure 7. In this experiment, the strain range of the test was limited to 0.08% in order to avoid damage to the silicon substrate. The resistance was measured for 10 s while holding the tensile strain at a constant level, and then the strain was applied again to evaluate the resistance change.

**Figure 8:**
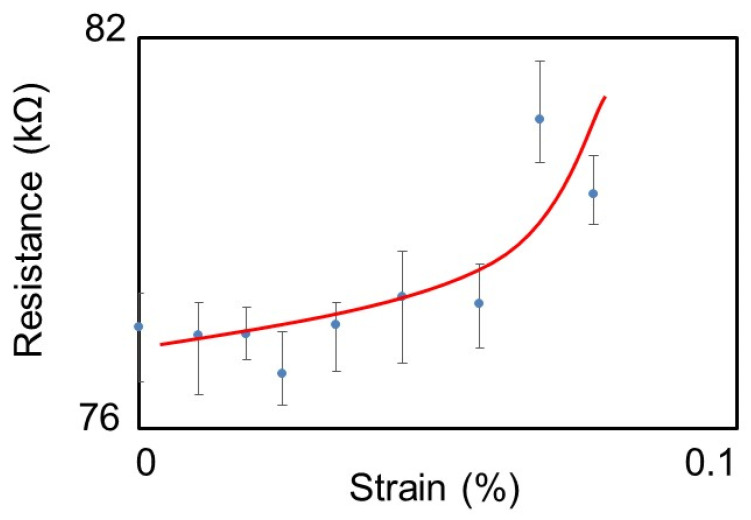
Strain-induced change of the electrical resistance of 50 nm-wide GNR.

The strain dependence of the measured resistance of GNR is shown in Figure 8. The error bars in the figure indicate the upper and lower resistance values during the measurement period. Although the variation of the measured values was not small, the resistance value clearly increased with increasing strain. The gauge factor calculated from the slope of the approximate line was about 50. The voltage and current characteristic analysis using the non-equilibrium Green’s function method showed that the band gap appears and the electrical resistance increased with increasing tensile strain in AGNR with metallic conduction properties [30]. The maximum gauge factor obtained in this analysis was about 70, and the measurement results agreed well with the theoretical analysis results. Therefore, the higher gauge factor than that of ordinary metal strain gauges (gauge factor: about 2) or that of conventional polysilicon (gauge factor: about ±30) could be attributed to the change in the band gap of the GNR due to tensile strain. In addition, the gauge factor was much larger than that of 10 μm-wide graphene (gauge factor: about 3.1 [23]), indicating that the utilization of a nanoribbon structure is effective for improving the sensitivity of graphene-based strain sensors. Although the strain sensitivity was not good in the region up to 0.05% strain, theoretical analysis has shown that sensitivity improvement in the micro-strain region can be achieved by increasing the length of the GNR and optimizing the measurement current or voltage [31]. Therefore, there is high potential for the development of high-sensitivity strain sensors by applying GNRs narrower than 50 nm.

## 4. Conclusions

In this study, we fabricated area-arrayed GNRs and evaluated the quality and strain dependence of their electrical conduction properties in order to realize a GNR-based strain sensor applicable in structural health monitoring. GNRs with widths ranging from 400 to 50 nm were fabricated by EB lithography between metal electrodes with 18 μm spacing. The I–V characteristics were evaluated for the fabricated GNRs. The current values of GNRs with widths of 400, 300, and 200 nm increased linearly with increasing applied voltage. It was confirmed that fabricated GNRs with a width of 200 nm or more exhibited metallic properties, and good ohmic contact was obtained between graphene and metal electrodes. The sheet resistance of GNRs was also evaluated based on the measurement results. The sheet resistances of GNRs with widths of 400 and 300 nm were 510–750 Ω/square, and the sheet resistance of GNRs with width of 200 nm was about 1000 Ω/square. The sheet resistance of GNRs with a width of 200 nm increased, but the GNR fabrication process developed in this study did not cause significant damage. On the other hand, for GNRs with a width of 50 nm, there were two types of GNRs: one showing metallic conduction in which the current value increased linearly with increasing applied voltage, and the other in which the current value increased nonlinearly. Theoretically, GNRs with ribbon widths of 100 nm or less are known to exhibit piezoresistive effects. Therefore, GNRs with a width of 50 nm were subjected to strain in a four-point bending test, and the strain dependence of electrical resistance was evaluated. As a result, a maximum gauge factor of about 50 was obtained in a strain range of 0.08% for GNRs showing metallic conductivity. This result indicated that GNRs could be applied to the development of highly sensitive strain sensors. Thus, we have successfully fabricated low-damage GNRs and demonstrated the possibility of developing highly sensitive strain sensors by applying GNRs.

## Data Availability

Data are contained within the article.
